# Evidence of Efficacy of the My Personal Health Guide Mobile Phone App on Antiretroviral Therapy Adherence Among Young African American Men Who Have Sex With Men at 1 Month: Randomized Controlled Trial

**DOI:** 10.2196/75005

**Published:** 2026-02-03

**Authors:** Mark S Dworkin, Kara Herrera, Sierra Upton, Casey M Luc, Jeb Jones, Paul Burns, Li Liu, Antonio Jimenez, Ruiqi Ren, Meaghan Woody, Robert Garofalo, Sangyoon Lee

**Affiliations:** 1 Community Outreach Intervention Projects University of Illinois Chicago Chicago, IL United States; 2 Rollins School of Public Health Emory University Atlanta United States; 3 John D. Bower School of Population Health University of Mississippi Medical Center Jackson, MS United States; 4 Adolescent and Young Adult Medicine Ann & Robert H. Lurie Children's Hospital of Chicago Chicago, IL United States; 5 Department of Computer Science Connecticut College New London, CT United States

**Keywords:** men who have sex with men, African American men who have sex with men, avatar-based mobile phone intervention, Black or African American, HIV, medical adherence, mobile apps, sexual and gender minorities, treatment adherence

## Abstract

**Background:**

Young African American men who have sex with men (AAMSM) experience disproportionately high HIV incidence and are less likely to achieve viral suppression compared to White men who have sex with men, an outcome that relies on antiretroviral therapy (ART) adherence. We created My Personal Health Guide, a talking relational agent–based mobile health app to improve ART adherence among young AAMSM.

**Objective:**

The objective was to determine the efficacy of My Personal Health Guide on improving ART adherence among young AAMSM living with HIV.

**Methods:**

We implemented a randomized controlled trial among young (aged 18-34 years) AAMSM with nonoptimal ART adherence throughout the United States between February 2020 and September 2023, predominantly through social media and by word of mouth, provider referral, and fliers in selected health care settings. Participants were randomized in a 1:1 ratio using permuted blocks of 8 to the intervention, My Personal Health Guide, or the attention control arm. ART adherence was assessed with Wilson’s 3-item self-reported adherence measurement and dichotomized at ≥80%. Logistic regression models using backward selection were used to evaluate the efficacy of My Personal Health Guide on ≥80% ART adherence at 1-month follow-up.

**Results:**

Among the 253 AAMSM at baseline, most (n=180, 71.1%) self-reported being ≥80% adherent to ART, over half (n=145, 57.3%) resided in the Southern United States, but all US regions were represented, nearly half (n=175, 42.3%) had some college education, over one-third (n=96, 37.9%) had less than optimal literacy, and approximately one-quarter (n=61, 24.1%) experienced housing insecurity in the past 6 months. The sample for analysis of the My Personal Health Guide app efficacy was 131 (intervention=76 and control=55). The odds of being ≥80% adherent to ART at 1-month follow-up were 3.97 (95% CI 1.26-12.55) times greater among participants randomized to the My Personal Health Guide app compared to the controls, after adjusting for ART adherence at baseline, treatment adherence self-efficacy, and ever being incarcerated. Additionally, for every 1-point increase in the HIV Treatment Adherence Self-Efficacy Scale, the odds of ≥80% ART adherence increased by 3% (odds ratio 1.03, 95% CI 1.00-1.06).

**Conclusions:**

Participants randomized to receive My Personal Health Guide reported nearly 4 times greater odds of being ≥80% adherent to ART compared to the attention control group at 1-month follow-up. To our knowledge, this is the first randomized controlled trial demonstrating improved medication adherence using a relational agent–based behavioral intervention. These findings provide evidence of short-term efficacy of My Personal Health Guide to improve ART adherence among young AAMSM. We recommend further research on the inclusion of relational agents in behavioral research, especially in populations affected by stigma and nonoptimal health literacy, where this nonhuman supportive and educational approach may be complementary to health care systems.

**Trial Registration:**

ClinicalTrials.gov NCT04217174; https://clinicaltrials.gov/study/NCT04217174

## Introduction

African American men who have sex with men (AAMSM) have disproportionate HIV infection rates, retention in care [1], mortality, and rates of viral nonsuppression [2-8], an outcome that relies on antiretroviral therapy (ART) adherence. Additionally, health literacy is a factor associated with ART adherence [9] that is lower among African American people than White people [10]. Therefore, interventions that bolster health literacy might improve healthy HIV-related behaviors like ART adherence, potentially resulting in personal and public health benefits.

Relational agents are computerized images that a person may react to as if they are in a relationship. A relational agent can be designed to promote healthy behavioral change. We developed My Personal Health Guide, an innovative theory-driven relational agent–based app with improvement of ART adherence as one of its objectives [11]. During iterative app development focus groups with young AAMSM living with HIV, participants reported universal acceptability for the African American realistic avatar we developed that spoke about, taught, explained, and encouraged behavior to promote healthy HIV-related outcomes [11]. Responding to meeting their adherence-related needs and interests, the app includes clear and simple language, an audiovisual format, and gamification. A pilot study performed in Chicago provided evidence that My Personal Health Guide has the potential to improve adherence [12]. Here, we present preliminary short-term follow-up results from a randomized controlled trial (RCT) of My Personal Health Guide that demonstrate evidence of improvement of ART adherence among young AAMSM living with HIV. We hypothesized that those randomized to the My Personal Health Guide app would have greater ART adherence compared to controls at 1-month follow-up post randomization. Evidence of short-term improvement in ART adherence provides scientific rationale for further research of this realistic avatar and relational agent approach as an adherence intervention and informs efforts to refine the approach to produce longer-term effects.

## Methods

### Intervention

My Personal Health Guide features a realistic talking avatar with supportive functions that were designed to inform users of HIV-related health information, encourage participants to take their medication, and facilitate adherence-related behavioral skills with reminders and push notifications to take medication and promotion of using weekly pill boxes. It also includes recordings of peers and health care providers offering personal and motivational messages. The app provides a private space, in one’s own mobile phone, for hearing and seeing information relevant to healthy living with HIV. Users may replay information, which can be especially helpful to people who are uncomfortable asking questions of their health care provider, including asking for a repeat of what was said or an explanation in simple terms.

### Design, Recruitment, Eligibility, and Enrollment

This RCT was performed among young AAMSM living with HIV. Participants were recruited throughout the United States between February 2020 and September 2023, predominantly through social media (eg, the dating app Jack’d and Facebook advertisements) and also by word of mouth, provider referral, and fliers in health care settings of the 3 enrollment sites: University of Illinois at Chicago, Emory University, and University of Mississippi Medical Center. Those who were eligible were assigned male sex at birth, currently identified as a man, African American or Black, aged 18-34 years, had at least one male sexual partner in their lifetime, English speaking, owned a smartphone, were initiating or prescribed oral ART, and had a history of nonoptimal ART adherence defined as having a detectable viral load within the past 4 weeks, self-reported nonoptimal ART adherence within the past 30 days, or were referred by a provider for adherence concerns. Those who were on injectable ART, participating in another HIV treatment study, or did not have a stable mailing address at the time of enrollment were ineligible for participation. This study was determined to be minimal risk, with no adverse events reported.

### Data Collection, Sample Size, and Randomization

**Figure 1:**
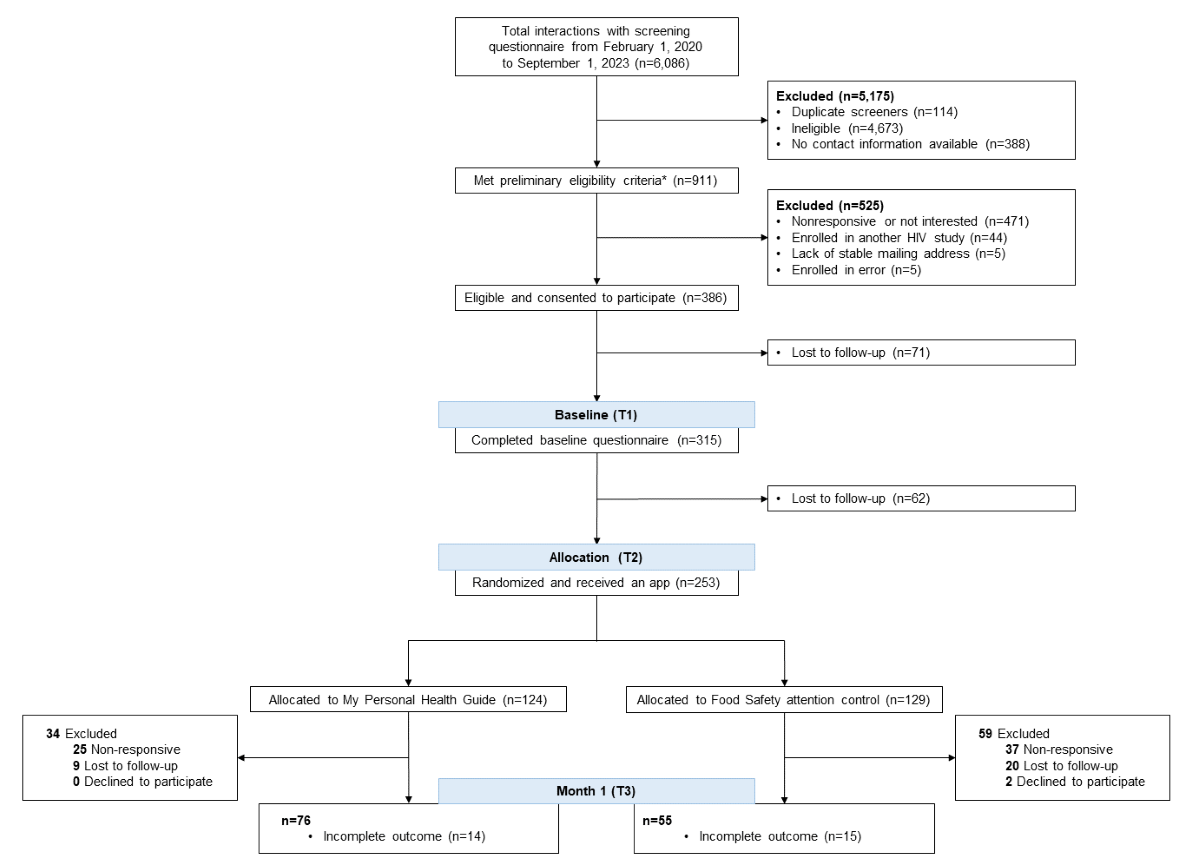
CONSORT (Consolidated Standards of Reporting Trials) diagram. Preliminary eligibility did not include verifying age and active HIV prescription.

Figure 1 summarizes participant interactions with the study through the first month of follow-up. A structured, interviewer-administered baseline questionnaire included collection of demographics, baseline knowledge of HIV (items taught in the app), self-reported adherence, incarceration history, housing stability, substance use history, self-efficacy (HIV Treatment Adherence Self-Efficacy Scale [HIV-ASES]) [13], literacy (adapted Rapid Estimate of Adult Literacy in Medicine-Short Form [REALM-SF]) [14], perceived social support (Multidimensional Scale of Perceived Social Support [MSPSS]) [15], and depression symptomatology (Patient Health Questionnaire for Depression [PHQ-9]) [16]. If a participant exhibited moderately severe or severe depressive symptomatology, they were encouraged to reach out to their provider for mental health resources. One month after the baseline interview, participants were scheduled for the app orientation and download that included explaining how to download, setting up a password, briefly pointing out features, and encouraging app use. Additionally, a check-in call was made monthly during 6 months of follow-up that asked troubleshooting questions about the app and collected self-reported ART adherence. Participants received compensation of US $40 for the baseline interview, US $75 for an appointment to download the app, and US $10 for responding to each monthly check-in call. Sample size calculations were carried out using Repeated Measures and Sample Size (RMASS2), the power calculation software for repeated measures design that allows attrition over time. The study was designed with a proposed sample size of 250 at the end of the study duration, or 295 at baseline, which would provide 80% statistical power to detect a 10% group difference in HIV care retention at the end of the study, considering a 15% attrition rate. Permuted randomization in blocks of 8 was used to allocate research participants in a 1:1 ratio to the intervention or control arm, and allocation concealment from the principal investigator, research staff, and research participants was carried out by the senior statistician. Participants randomized to the intervention arm received the My Personal Health Guide app, and those randomized to the attention control arm received a food safety app, which featured an avatar and functions related to food and nutrition, narrated illustrated stories about food safety, an educational game about sugar content in food, and information promoting oral health. The principal investigator and research participants were blinded after assignment to the intervention. Research coordinators oriented the participants to their respective app after randomization.

### Outcome Measurement

ART adherence was assessed with Wilson’s 3-item self-reported adherence measurement, which has been validated against electronic drug monitoring for HIV medication (Cronbach α=0.83) [17]. These three questions include (1) “In the last 30 days, on how many days did you miss at least one dose of any of your HIV medication?” (with a response range from 0 to 30 days); (2) “In the last 30 days, how often did you take your HIV medication in the way you were supposed to?” (with response options including Never, Rarely, Sometimes, Usually, Almost Always, and Always); and (3) “In the last 30 days, how good a job did you do at taking your HIV medications in the way you were supposed to?” (with response options including Very Poor, Poor, Fair, Good, Very Good, and Excellent). Answers were averaged together and converted to a percentage score on a 100-point scale. Consistent with other studies [18,19], 80% was defined as the cut point for nonoptimal ART adherence. HIV viral load was the intended primary outcome for this research. However, the trial was disturbed by the COVID-19 pandemic, and significant difficulty occurred collecting viral load from this population. Therefore, self-reported ART adherence was used.

### Statistical Analysis

The primary statistical goal for this analysis was to determine the efficacy of the My Personal Health Guide app on ART adherence at 1-month follow-up. Participant demographic and study characteristics are presented as means and SDs for continuous variables and as absolute numbers (n) and proportions (%) for categorical variables stratified by randomization arm. The success of randomization was assessed using 2-tailed *t* tests for continuous baseline characteristics or chi-square tests for categorical characteristics. Baseline characteristics between individuals that completed both the baseline meeting and 1-month follow-up versus those who had missing data at 1-month follow-up were compared to ensure comparability between the samples. Bivariate comparisons were made between participant characteristics and ART adherence at baseline (≥80% vs <80%) using chi-square tests for categorical data, Cochran-Armitage tests for ordinal data, and independent *t* tests for continuous data. Logistic regression models with the backward model selection method were used to evaluate the effect of the My Personal Health Guide app, adjusted for significant factors that were associated with ART adherence at month 1, with a *P*≤.05 considered statistically significant. Baseline adherence was adjusted for a priori. The baseline characteristics that were considered in the logistic regression models included age, ethnicity, remote participation, residence, education, employment status, sexual orientation, currently in a committed relationship, health literacy (REALM-SF), HIV-ASES, depression symptomatology (PHQ-9), perceived social support (MSPSS), ever incarcerated, housing insecurity, and substance use seriousness (serious includes cocaine, methamphetamines, crack, heroin, and opiates; less serious includes alcohol, marijuana, ecstasy, speedballs, and gamma-hydroxybutyrate; none includes no use of substances or alcohol) at baseline. Adjusted model results are presented as odds ratios (ORs) and 95% CI.

### Ethical Considerations

This research was reviewed and approved by the institutional review boards of the University of Illinois Chicago, Emory University, and the University of Mississippi Medical Center (protocol #2019-1184). The research involving human data was conducted in accordance with institutional guidelines of the University of Illinois Chicago, Emory University, and the University of Mississippi Medical Center. All participants were administered written informed consent. All data were deidentified prior to analysis and dissemination. Participants were compensated up to US $125 by the 1-month follow-up by their preferred method of check, cash, or e-code.

## Results

### Sample Characteristics

We attempted to contact the 911 men that met preliminary eligibility criteria, responded to recruitment methods, and provided contact information. Among those, 471 were either not responsive or not interested in the study, 44 were enrolled in another HIV treatment study, and 5 did not have a stable mailing address in order to receive study materials. Five participants were erroneously enrolled in the study due to either being unable to verify having a current HIV prescription (n=3) or having been previously enrolled in the study (n=2). A total of 386 men were consented. Among these, the baseline questionnaire was completed by 315 (81.6%), and 253 (65.5%) had the study apps installed on their mobile phones 1 month later (Figure 1), including 124 (49%) randomized to My Personal Health Guide and 129 (51%) randomized to the Food Safety attention control app. After 1 month, 93 participants did not respond to contact attempts to complete the month 1 check-in call and were excluded from the current analysis. The remaining 160 participants completed the first check-in phone call, and of those, 131 had complete self-reported adherence data at month 1.

The mean age of the 253 participants at baseline was 29.4 (SD 3.7; range18-35) years; most (n=221, 87.4%) were 25-34 years of age. Few (n=18, 7%) identified as Hispanic. Most participated remotely (n=202, 79.8%). Most participants were enrolled by the University of Illinois at Chicago (n=131, 51.8%) and Emory University (n=106, 41.9%), and 16 (6%) were enrolled by the University of Mississippi Medical Center. Over half (n=145, 57.3%) of participants resided in the Southern United States, but all US regions were represented in the sample. Nearly half (n=175, 42.3%) had some college education, and over half (n=146, 57.7%) reported being employed. Three-quarters (n=180, 71.1%) identified as homosexual or gay and were not currently in a committed relationship with a male partner (n=198, 78.3%). Over one-third (n=96, 38%) had less than optimal literacy (adapted REALM-SF). Over half reported mild-severe depressive symptomatology, with 26% (n=65) reporting mild (PHQ-9=5.0-9.9), 14% (n=35) reporting moderate (PHQ-9=10.0-14.9), 9% (n=23) reporting moderately severe (PHQ-9=15.0-19.9), and 4% (n=11) reporting severe (PHQ-9>20.0) depressive symptomatology. The median HIV-ASES score was 104 (IQR 89-114). Although most (n=150, 59.3%) reported high perceived social support (MSPSS>5.0), 9% (n=23) had low (MSPSS<3.0) perceived social support, and 32% (n=80) had moderate (MSPSS=3.0-5.0) perceived social support. Approximately one-quarter experienced housing insecurity in the past 6 months (n=61, 24%), and nearly one-third were ever incarcerated (n=77, 30%). Some (n=39, 15%) reported serious substance use in the past 2 weeks. Nearly three-quarters (n=178, 70.4%) reported less serious substance use, including alcohol, marijuana, ecstasy, speedballs, or gamma-hydroxybutyrate. Most (n=180, 71.1%) self-reported being ≥80% adherent to ART at baseline. Group comparisons for all baseline characteristics and ART adherence at baseline (Table 1) revealed that randomizations were successful.

**Table 1:** Baseline demographic characteristics and antiretroviral therapy (ART) adherence of young African American men who have sex with men living with HIV stratified by randomization arm (n=253), 2020-2024.

		Overall (n=253)	Intervention (n=124)	Control (n=129)	*P* value	
Age in years, mean (SD)		29.4 (3.7)	29.6 (3.5)	29.1 (4)	.46^a^	
**Age group (years), n (%)**						.41^b^
	18-24	32 (12.6)	13 (10.5)	19 (14.7)		
	25-34	221 (87.4)	111 (89.5)	110 (85.3)		
**Ethnicity, n (%)**						>.99^b^
	Hispanic	18 (7.1)	9 (7.3)	9 (7)		
	Not Hispanic	235 (92.9)	115 (92.7)	120 (93)		
**Enrollment site, n (%)**						.18^b^
	University of Illinois at Chicago	131 (51.8)	59 (47.6)	72 (55.8)		
	Emory University	106 (41.9)	54 (43.5)	52 (40.3)		
	University of Mississippi Medical Center	16 (6.3)	11 (8.9)	5 (3.9)		
**Remote participation, n (%)**						.87^b^
	Yes	202 (79.8)	98 (79)	104 (80.6)		
	No	51 (20.2)	26 (21)	25 (19.4)		
**Residence, n (%)**						.42^c^
	Midwest	77 (30.4)	34 (27.4)	43 (33.3)		
	Northeast	27 (10.7)	12 (9.7)	15 (11.6)		
	South	145 (57.3)	77 (62.1)	68 (52.7)		
	West	4 (1.6)	1 (0.8)	3 (2.3)		
**Education, n (%)**						.59^b^
	College	68 (26.9)	30 (24.2)	38 (29.5)		
	Some college	107 (42.3)	51 (41.1)	56 (43.4)		
	High school or GED^d^	65 (25.7)	36 (29)	29 (22.5)		
	Less than high school	13 (5.1)	7 (5.6)	6 (4.7)		
**Employment, n (%)**						.45^b^
	Active duty	1 (0.4)	0 (0)	1 (0.8)		
	Employed	146 (57.7)	75 (60.5)	71 (55)		
	Student	18 (7.1)	10 (8.1)	8 (6.2)		
	Unable to work or unemployed	83 (32.8)	36 (29)	47 (36.4)		
	Missing	5 (2)	3 (2.4)	2 (1.6)		
**Sexual orientation, n (%)**						.81^b^
	Homosexual or gay	180 (71.1)	85 (68.5)	95 (73.6)		
	Bisexual	49 (19.4)	27 (21.8)	22 (17.1)		
	Heterosexual or straight	4 (1.6)	2 (1.6)	2 (1.6)		
	Other	20 (7.9)	10 (8.1)	10 (7.8)		
**Currently in a committed relationship with a male partner, n (%)**						.66^b^
	Yes	55 (21.7)	25 (20.2)	30 (23.3)		
	No	198 (78.3)	99 (79.8)	99 (76.7)		
**Marital status, n (%)**						.69^b^
	Legally married	10 (4)	4 (3.2)	6 (4.7)		
	Registered domestic partnership	1 (0.4)	1 (0.8)	0 (0)		
	Widowed	1 (0.4)	0 (0)	1 (0.8)		
	Divorced	8 (3.2)	5 (4)	3 (2.3)		
	Separated	5 (2)	2 (1.6)	3 (2.3)		
	Never married	228 (90.1)	112 (90.3)	116 (89.9)		
**Health literacy (adapted REALM-SF^e^** **), n (%)**						>.99^b^
	Nonoptimal (at least 1 wrong)	96 (37.9)	47 (37.9)	49 (38)		
	Optimal (none wrong)	157 (62.1)	77 (62.1)	80 (62)		
PHQ-9^f^ score, mean (SD)		6.67 (5.81)	6.71 (5.96)	6.62 (5.69)	.90^a^	
**PHQ-9 score, n (%)**						.72^g^
	None or minimal (<5.0)	117 (46.2)	55 (44.4)	62 (48.1)		
	Mild (5.0-9.9)	65 (25.7)	35 (28.2)	30 (23.3)		
	Moderate (10.0-14.9)	35 (13.8)	14 (11.3)	21 (16.3)		
	Moderately severe (15.0-19.9)	23 (9.1)	11 (8.9)	12 (9.3)		
	Severe (≥20.0)	11 (4.3)	7 (5.6)	4 (3.1)		
	Missing	2 (0.8)	2 (1.6)	0 (0)		
**HIV-ASES^h^** **, n (%)**						.29^a^
	Mean (SD)	98.1 (20.2)	99.5 (18.1)	96.8 (22)		
	Median (IQR)	104 (89.0-114)	105 (88.3-114)	103 (89.0-115)		
	Missing, n (%)	3 (1.2)	2 (1.6)	1 (0.8)		
MSPSS^i^ score, mean (SD)		5.08 (1.36)	5.09 (1.45)	5.08 (1.28)	.98^a^	
**MSPSS score, n (%)**						
	Low (<3.0)	23 (9.1)	13 (10.5)	10 (7.8)	.68^b^	
	Moderate (3.0-5.0)	80 (31.6)	37 (29.8)	43 (33.3)	.81^d^	
	High (≥5.1)	150 (59.3)	74 (59.7)	76 (58.9)		
**Ever incarcerated, n (%)**						.07^b^
	Yes	77 (30.4)	45 (36.3)	32 (24.8)		
	No	176 (69.6)	79 (63.7)	97 (75.2)		
**Housing insecurity** **in previous 6 months, n (%)**						.64^b^
	Yes	61 (24.1)	32 (25.8)	29 (22.5)		
	No	192 (75.9)	92 (74.2)	100 (77.5)		
**Substance use seriousness in previous 2 weeks** ^j^ **, n (%)**						.32^b^
	None	36 (14.2)	17 (13.7)	19 (14.7)		
	Less serious	178 (70.4)	92 (74.2)	86 (66.7)		
	Serious	39 (15.4)	15 (12.1)	24 (18.6)		
**Adherence to ART (baseline), n (%)**						.14^b^
	Yes (≥80)	180 (71.1)	94 (75.8)	86 (66.7)		
	No (<80)	73 (28.9)	30 (24.2)	43 (33.3)		
**Adherence to ART (month 1), n (%)**						.04^b^
	Yes (≥80)	111 (43.9)	69 (55.6)	42 (32.6)		
	No (<80)	20 (7.9)	7 (5.6)	13 (10.1)		
**Missing data (month 1), n (%)**						.005^b^
	Yes	122 (48.2)	48 (38.7)	74 (57.4)		
	No	131 (51.8)	76 (61.3)	55 (42.6)		

^a^Independent samples *t* test.

^b^Chi-square test for comparison.

^c^Fisher exact test.

^d^GED: General Educational Development.

^e^REALM-SF: Rapid Estimate of Adult Literacy in Medicine-Short Form.

^f^PHQ-9: Patient Health Questionnaire for Depression.

^g^Cochran-Armitage test.

^h^HIV-ASES: HIV Treatment Adherence Self-Efficacy Scale.

^i^MSPSS: Multidimensional Scale of Perceived Social Support.

^j^Serious includes cocaine, methamphetamines, crack, heroin, opiates; less serious includes alcohol, marijuana, ecstasy, speedballs, gamma-hydroxybutyrate.

### Primary Outcome

Among the 253 participants who were randomized and downloaded an app, 131 (51.8%) completed the 1-month follow-up for ART adherence, and 122 (48.2%) did not. A significantly greater proportion of those randomized to the Food Safety app were missing 1-month follow-up data compared to the My Personal Health Guide app (74/129, 57.4% vs 48/124, 38.7%; *P*=.005; Table 2). Additionally, remote participation, employment, substance use, and ART adherence at baseline differed by complete 1-month follow-up data and were therefore considered during multivariable model construction. Bivariate analysis (Table 3) revealed the mean HIV-ASES score (mean 103, SD 15.8 vs mean 86.4, SD 28.5; *P*=.02) was significantly higher in those who had ≥80% adherence to ART at 1 month as compared to those who had <80% adherence at 1 month. The mean depression symptomatology scale (PHQ-9) was moderately significantly lower among those who were ≥80% adherent to ART at 1 month as compared to those who were <80% adherent to ART at 1 month (5.70, SD 4.96 vs 9.00, SD 7.43; *P*=.07). There was also a significantly higher prevalence of being in a committed relationship with a male partner among those who had ≥80% adherence to ART at 1 month as compared to those who had <80% adherence at 1 month (25/111, 22.5% vs 0/20, 0%; *P*=.01).

In multivariable analysis (Table 4), the odds of being ≥80% adherent to ART at 1-month follow-up were almost 4 times greater (OR 3.97, 95% CI 1.26-12.55) among participants randomized to the My Personal Health Guide app as compared to the Food Safety app after adjusting for other characteristics that were also associated with ART adherence at 1 month, including HIV-ASES and having a history of ever being incarcerated. Every 1-point increase in the HIV-ASES increased the odds of ≥80% ART adherence by 3% (OR 1.03, 95% CI 1.00-1.06). A history of ever being incarcerated reduced the odds of ≥80% ART adherence (OR 0.31, 95% CI 0.09-1.06). ART adherence at baseline was marginally associated with ≥80% ART at 1-month follow-up (OR 2.97, 95% CI 0.88-9.99).

**Table 2:** Baseline demographic characteristics and antiretroviral therapy (ART) adherence of young African American men who have sex with men living with HIV stratified by missing outcome (self-reported ART adherence at 1 month) data (n=253), 2020-2024.

		Overall (n=253)	Missing outcome data (month 1; n=122)	Complete outcome data (month 1; n=131)	*P* value	
Age in years, mean (SD)		29.4 (3.7)	29.2 (3.6)	29.5 (3.6)	.51^a^	
**Age group (years), n (%)**						.98^b^
	18-24	32 (12.6)	16 (13.1)	16 (12.2)		
	25-34	221 (87.4)	106 (86.9)	115 (87.8)		
**Ethnicity, n (%)**						.37^b^
	Hispanic	18 (7.1)	11 (9)	7 (5.3)		
	Not Hispanic	235 (92.9)	111 (91)	124 (94.7)		
**Enrollment site, n (%)**						.28^b^
	University of Illinois at Chicago	131 (51.8)	66 (54.1)	65 (49.6)		
	Emory University	106 (41.9)	46 (37.7)	60 (45.8)		
	University of Mississippi Medical Center	16 (6.3)	10 (8.2)	6 (4.6)		
**Remote participation, n (%)**						<.001^b^
	Yes	202 (79.8)	83 (68)	119 (90.8)		
	No	51 (20.2)	39 (32)	12 (9.2)		
**Residence, n (%)**						
	Midwest	77 (30.4)	44 (36.1)	33 (25.2)	.16^b^	
	Northeast	27 (10.7)	15 (12.3)	12 (9.2)	.14^c^	
	South	145 (57.3)	61 (50)	84 (64.1)		
	West	4 (1.6)	2 (1.6)	2 (1.5)		
**Education, n (%)**						.29^b^
	College	68 (26.9)	26 (21.3)	42 (32.1)		
	Some college	107 (42.3)	56 (45.9)	51 (38.9)		
	High school or GED^d^	65 (25.7)	33 (27)	32 (24.4)		
	Less than high school	13 (5.1)	7 (5.7)	6 (4.6)		
**Employment, n (%)**						.005
	Active duty	1 (0.4)	0 (0)	1 (0.8)		
	Employed	146 (57.7)	58 (47.5)	88 (67.2)		
	Student	18 (7.1)	12 (9.8)	6 (4.6)		
	Unable to work or unemployed	83 (32.8)	49 (40.2)	34 (26)		
	Missing	5 (2)	3 (2.5)	2 (1.5)		
**Sexual orientation, n (%)**						.87^b^
	Homosexual or gay	180 (71.1)	84 (68.9)	96 (73.3)		
	Bisexual	49 (19.4)	25 (20.5)	24 (18.3)		
	Heterosexual or straight	4 (1.6)	2 (1.6)	2 (1.5)		
	Other	20 (7.9)	11 (9)	9 (6.9)		
**Currently in a committed relationship with a male partner, n (%)**						.36^b^
	Yes	55 (21.7)	30 (24.6)	25 (19.1)		
	No	198 (78.3)	92 (75.4)	106 (80.9)		
**Marital status, n (%)**						
	Legally married	10 (4)	4 (3.3)	6 (4.6)	.71^b^	
	Registered domestic partnership	1 (0.4)	0 (0)	1 (0.8)	.83^c^	
	Widowed	1 (0.4)	0 (0)	1 (0.8)		
	Divorced	8 (3.2)	5 (4.1)	3 (2.3)		
	Separated	5 (2)	2 (1.6)	3 (2.3)		
	Never married	228 (90.1)	111 (91)	117 (89.3)		
**Health literacy (adapted REALM-SF^e^** **), n (%)**						.41^b^
	Nonoptimal (at least 1 wrong)	96 (37.9)	50 (41)	46 (25.1)		
	Optimal (None wrong)	157 (62.1)	72 (59)	85 (64.9)		
PHQ-9^f^ score, mean (SD)		6.67 (5.81)	7.16 (6.10)	6.21 (5.51)	.20^a^	
**PHQ-9 score, n (%)**						.78^b^
	None or minimal (<5.0)	117 (46.2)	55 (45.1)	62 (47.3)		
	Mild (5.0-9.9)	65 (25.7)	29 (23.8)	36 (27.5)		
	Moderate (10.0-14.9)	35 (13.8)	18 (14.8)	17 (13)		
	Moderately severe (15.0-19.9)	23 (9.1)	12 (9.8)	11 (8.4)		
	Severe (≥20.0)	11 (4.3)	7 (5.7)	4 (3.1)		
	Missing	2 (0.8)	1 (0.8)	1 (0.8)		
**HIV-ASES^g^** **score**						.11^a^
	Mean (SD)	98.1 (20.2)	96 (21.1)	100 (19.2)		
	Median (IQR)	104 (89-114)	105 (90-115)	104 (89-114)		
	Missing, n (%)	3 (1.2)	0 (0)	3 (2.3)		
MSPSS^h^ score, mean (SD)		5.08 (1.36)	4.96 (1.39)	5.19 (1.33)	.18^a^	
**MSPSS score, n (%)**						
	Low (<3.0)	23 (9.1)	11 (9)	12 (9.2)	.12^b^	
	Moderate (3.0-5.0)	80 (31.6)	46 (37.7)	34 (26)	.17^i^	
	High (≥5.1)	150 (59.3)	65 (53.3)	85 (64.9)		
**Ever incarcerated, n (%)**						.14^b^
	Yes	77 (30.4)	43 (35.2)	34 (26)		
	No	176 (69.6)	79 (64.8)	97 (74)		
**Housing insecurity in previous 6 months**, n (%)						.57^b^
	Yes	61 (24.1)	27 (22.1)	34 (26)		
	No	192 (75.9)	95 (77.9)	97 (74)		
**Substance use seriousness in previous 2 weeks^j^** **, n (%)**						.02^b^
	None	36 (14.2)	25 (20.5)	11 (8.4)		
	Less serious	178 (70.4)	77 (63.1)	101 (77.1)		
	Serious	39 (15.4)	20 (16.4)	19 (14.5)		
**Adherence to ART (baseline), n (%)**						.05^b^
	Yes (≥80%)	180 (71.1)	79 (64.8)	101 (77.1)		
	No (<80%)	73 (28.9)	43 (35.2)	30 (22.9)		

^a^Independent samples *t* test.

^b^Chi-square test for comparison.

^c^Fisher exact test.

^d^GED: General Educational Development.

^e^REALM-SF: Rapid Estimate of Adult Literacy in Medicine-Short Form.

^f^PHQ-9: Patient Health Questionnaire for Depression.

^g^HIV-ASES: HIV Treatment Adherence Self-Efficacy Scale.

^h^MSPSS: Multidimensional Scale of Perceived Social Support.

^i^Cochran-Armitage test.

^j^Serious includes cocaine, methamphetamines, crack, heroin, opiates; less serious includes alcohol, marijuana, ecstasy, speedballs, gamma-hydroxybutyrate.

**Table 3:** Bivariate analysis of demographic characteristics of young African American men who have sex with men living with HIV by antiretroviral therapy (ART) adherence at 1 month (n=131), 2020-2024.

												Overall (n=131)		<80% ART adherence (n=20)		≥80 ART adherence (n=111)		*P* value	
**Randomization, n (%)**																			.03^a^
	My Personal Health Guide (intervention)										76 (58)		7 (35)		69 (62.2)				
	Food safety (control)										55 (42)		13 (65)		42 (37.8)				
Age (years), mean (SD)												29.5 (3.62)		29.9 (2.60)		29.4 (3.78)		.53^b^	
**Age group (years), n (%)**																			.13^a^
		25-34									115 (87.8)		20 (100)		95 (85.6)				
		18-24									16 (12.2)		0 (0)		16 (14.4)				
**Ethnicity, n (%)**																			.59^a^
							Hispanic				7 (5.3)		0 (0)		7 (6.3)				
							Not Hispanic				124 (94.7)		20 (100.0)		104 (93.7)				
**Remote participation, n (%)**																			.39^a^
			Yes								119 (90.8)		17 (85.0)		102 (91.9)				
			No								12 (9.2)		3 (15.0)		9 (8.1)				
**Residence, n (%)**																			.63^a^
	Midwest										33 (25.2)		7 (35.0)		26 (23.4)				
	Northeast										12 (9.2)		2 (10.0)		10 (9.0)				
	South										84 (64.1)		11 (55.0)		73 (65.8)				
	West										2 (1.5)		0 (0.0)		2 (1.8)				
**Education, n (%)**																			.75^a^
				College							68 (26.9)		18 (26.5)		50 (73.5)				
									Some college		107 (42.3)		30 (28.0)		77 (72.0)				
									High school or GED^c^		65 (25.7)		22 (33.8)		43 (66.2)				
									Less than high school		13 (5.1)		3 (23.1)		10 (76.9)				
**Employment, n (%)**																			.27^a^
										Employed, student, active duty		95 (72.5)		12 (60.0)		83 (74.8)			
										Unemployed or unable to work		34 (26.0)		7 (35.0)		27 (24.3)			
										Missing		2 (1.5)		1 (5)		1 (0.9)			
**Sexual orientation, n (%)**																			.44^a^
					Homosexual or gay						96 (73.3)		18 (90)		78 (70.3)				
					Bisexual						24 (18.3)		2 (10)		22 (19.9)				
					Heterosexual or straight						2 (1.5)		0 (0)		2 (1.9)				
					Other						9 (6.9)		0 (0)		9 (8.1)				
**Currently in a committed relationship with a male partner, n (%)**																			.01^a^
								Yes			25 (19.1)		0 (0)		25 (22.5)				
								No			106 (80.9)		20 (100)		86 (77.5)				
**Health literacy (adapted REALM-SF^d^** **), n (%)**																			>.99^a^
						Nonoptimal (at least 1 wrong)					46 (35.1)		7 (35)		39 (35.1)				
						Optimal (none wrong)					85 (64.9)		13 (65)		72 (64.9)				
PHQ-9^e^ score, mean (SD)												6.21 (5.51)		9 (7.43)		5.70 (4.96)		.07^b^	
PHQ-9 (missing), n (%)												1 (0.8)		0 (0)		1 (0.9)		.07^b^	
HIV-ASES^f^ score, mean (SD)												100 (19.2)		86.4 (28.5)		103 (15.8)		.02^b^	
HIV-ASES (missing), n (%)												3 (2.3)		0 (0)		3 (2.7)		02^b^	
MSPSS^g^ score, mean (SD)												5.19 (1.33)		4.81 (1.36)		5.26 (1.32)		.18^b^	
**Ever incarcerated**																			.41^a^
							Yes				34 (26)		7 (35)		27 (24.3)				
							No				97 (74)		13 (65)		84 (75.7)				
**Housing insecurity** **in previous 6 months**																			.41^a^
										Yes		34 (26)		7 (35)		27 (24.3)			
										No		97 (74)		13 (65)		84 (75.7)			
**Substance use seriousness in previous 2 weeks^h^**																			.12^a^
						Serious					19 (14.5)		6 (30)		13 (11.7)				
						Less serious					101 (77.1)		13 (65)		88 (79.3)				
						None					11 (8.4)		1 (5)		10 (9)				

^a^Fisher exact test.

^b^Independent samples *t* test.

^c^GED: General Educational Development.

^d^REA LM-SF: Rapid Estimate of Adult Literacy in Medicine-Short Form.

^e^PHQ-9: Patient Health Questionnaire for Depression.

^f^HIV-ASES: HIV Treatment Adherence Self-Efficacy Scale.

^g^MSPSS: Multidimensional Scale of Perceived Social Support.

^h^Serious includes cocaine, methamphetamines, crack, heroin, opiates; less serious includes alcohol, marijuana, ecstasy, speedballs, gamma-hydroxybutyrate

**Table 4:** Multivariable logistic regression for the association between My Personal Health Guide and ≥80% antiretroviral therapy (ART) adherence at 1-month follow-up among young African American men who have sex with men living with HIV (n=131), 2020-2024.

					Unadjusted odds ratio		Adjusted odds ratio (95% CI)		*P* value
**Randomization**									.02
	My Personal Health Guide (intervention)				4.55		3.97 (1.26-12.55)		
	Food Safety (control)				Reference		Reference		
**ART adherence at baseline**									.08
		≥80%		3.05		2.97 (0.88-9.99)			
		<80%		Reference		Reference			
HIV-ASES^a^					1.04		1.03 (1.00-1.06)		.04
**Ever incarcerated**									.06
			Yes	0.60		0.31 (0.09-1.06)			
			No	Reference		Reference			

^a^HIV-ASES: HIV Treatment Adherence Self-Efficacy Scale.

## Discussion

### Principal Findings

Participants randomized to receive My Personal Health Guide demonstrated nearly 4 times greater odds of being ≥80% adherent to ART compared to participants who received the Food Safety control app at 1-month follow-up. This is a substantial magnitude, and although these are short-term follow-up results, these data provide strong evidence that a relational agent approach to improving medication adherence is promising.

Our study is the first of its kind in the field of HIV medication adherence. We are aware of only 2 other published RCTs of a relational agent for behavior change. Rubin et al [20] performed an RCT of a relational agent to deliver a brief alcohol intervention and referral to treatment to veterans within VA primary care clinics. Their approach involved 2 meetings with the relational agent, who requested a commitment to change. They reported that their relational agent elicited concern about the consequences of drinking and bolstered motivation to change. Although they did not observe accelerated decline in drinking during 3 months with their intervention compared to their control group, they noted that their intervention group was more likely to receive referrals for follow-up and that this approach allowed for more screening and brief intervention without increasing the burden on clinic staff. Another study by Prochaska et al [21] studied a smartphone intervention called Woebot for substance use disorders that used a therapeutic relational agent delivering cognitive behavioral therapy. At 8 weeks, they reported reduced substance use occasions compared to a waitlist group. These studies demonstrate the potential for a relational agent to influence behavior and underscore the scientific rationale for their application to the field of medication adherence. Relational mobile health interventions may be particularly beneficial for younger (18-24 years) AAMSM because they are more likely to still be developing medication-taking routines and may be especially receptive to interactive mobile technologies that can bolster daily ART adherence (Hightow-Weidman et al [22]). Given that these studies and ours are RCTs, they provide evidence for consideration of relational agents in the design of behavioral interventions, especially involving conditions where stigma about diagnosis or treatment could create hesitancy with face-to-face health care provider education or counseling.

In addition to our study demonstrating a large magnitude efficacy, there are several other strengths and considerations. First, our study began shortly before the COVID-19 pandemic spread throughout the United States, disrupting in-person research with lockdowns and other restrictions. This led to a nearly entirely remote rather than facility-based administration and may have made the meeting with research staff for orientation to the app less interactive than it might have been otherwise. The remote approach could have diminished the participant’s feeling of accountability to the study or facilitated participation by reducing the barrier of travel to a study activity. Second, unlike medication or immunization, our app intervention involves a passive approach. In other words, patients are encouraged to use it, but it is their choice how much of the app they experience and how often they open it. The app was designed to have new functions and experiences occur with repeated use to encourage return to the app. However, we have no control to ensure regular app exposure. Despite these issues, we observed a significant effect in achieving the adherence goal. This suggests that the effect might increase further and benefit more patients with additional app refinement and involvement of health care providers or case managers in implementation who could refer the app to their patients and check in with them at future appointments. Third, our study sample was diverse, including a large proportion who experienced housing insecurity, screened for moderate or severe depression, and reported a history of incarceration. Additionally, our sample included an overrepresentation of men living in the Southern United States, a high HIV incidence region where many experience structural barriers to HIV care [23] and where a low-cost, scalable intervention like ours could contribute to achieving higher prevalence of viral suppression and help end the HIV epidemic. Finally, our study also demonstrated an independent effect of self-efficacy on being ≥80 adherent. This finding is consistent with other studies, such as those by Dworkin et al [24], Barclay et al [25], and Waldrop-Valverde et al [26]. However, this is the first study to report this specifically in a population of young AAMSM living with HIV.

### Limitations

Limitations of this study included that many participants were recruited using a social media dating app and the responsiveness of participants to the 1-month call was moderate, both of which may limit generalizability. Notably, we struggled to recruit younger (18-24 years) AAMSM; therefore, we recommend future studies use a multimodal recruitment approach. Additionally, missingness of self-reported ART adherence was high, and we observed a higher proportion of those randomized to the control with missing data at month 1. Study procedures were disrupted by the COVID-19 pandemic, necessitating the change from a facility-based trial to online. While this pivot allowed for further recruitment and retention, it compromised the collection of viral load data from participants’ clinics, which was initially an outcome of interest. This change also impacted the collection of dried blood spots at the 6-month follow-up, which was initially intended to be conducted on-site and changed to be self-administered in the participant’s home to accommodate COVID-19 stay-at-home orders. This resulted in a high proportion of missingness. Additionally, the laboratory methodology for the threshold of detection changed during the study period and used a higher threshold (<839 copies/mL) than the clinical definition of undetectable viral load (<200 copies/mL). Similarly, the usage of Wisepill devices to monitor ART adherence was low, so self-report was used as the primary outcome of interest. Using self-reported ART adherence also had limitations. Self-report can overestimate individual medication-taking behavior and therefore may introduce recall and social desirability bias. However, self-reported adherence is often used to assess ART adherence in both routine care and research settings and has been correlated with viral load and other objective markers of adherence [26]. Last, these findings are limited to the first month of observation. Additional follow-up analysis from this trial is planned to determine evidence of long-term effect.

### Conclusion

These findings provide evidence that My Personal Health Guide has short-term efficacy in improving ART adherence among young AAMSM living with HIV and contribute to the emerging field of technology-based behavioral interventions promoting health using relational agents. We recommend further research on the inclusion of relational agents in behavioral research, especially in populations affected by stigma and nonoptimal health literacy, where this nonhuman supportive and educational approach may be complementary to health care systems.

## Appendix

Multimedia Appendix 1 CONSORT 2010 checklist.

## Abbreviations

AAMSM: African American men who have sex with men
ART: antiretroviral therapy
HIV-ASES: HIV Treatment Adherence Self-Efficacy Scale
MSPSS: Multidimensional Scale of Perceived Social Support
OR: odds ratio
PHQ-9: Patient Health Questionnaire for Depression
RCT: randomized controlled trial
REALM-SF: Rapid Estimate of Adult Literacy in Medicine-Short Form
RMASS2: Repeated Measures and Sample Size

## Data Availability

A deidentified dataset, a data dictionary defining each field in the set, the study protocol including the statistical analysis plan, and informed consent forms will be made available upon request and with publication acceptance. Per university protocol, all sensitive and high-risk data will be transmitted via an encrypted protocol, or Secure File Transfer Protocol, to individuals outside of the university with investigator support, approval from the data safety and monitoring board, and a signed data access agreement.
